# Rashba Metamaterials and Metasurfaces with Zero Reflectivity and Effect of Surface States in Ultrathin Metal Films

**DOI:** 10.1021/acsami.3c15285

**Published:** 2024-01-17

**Authors:** Fedor Kusmartsev, Binglei Zhang, Yang Liu, Yi Luo, James Vincent-Ward, Fatemah Alkallas, Amira Ben Gouider Trabelsi, Anna Kusmartseva

**Affiliations:** † College of Engineering and Physical Sciences, Khalifa University, P.O. Box 127788 Abu Dhabi 51133, United Arab Emirates; ‡ Microsystem and Terahertz Research Center, Chengdu 610200, P. R. China; § Department of Physics, Loughborough University, Loughborough LE11 3TU, U.K.; ∥ Department of Physics, College of Science, Princess Nourah Bint Abdulrahman University, P.O. Box 84428, Riyadh 11671, Saudi Arabia

**Keywords:** Rashba spin orbit coupling, metamaterials, metasurfaces, zero reflectivity, surface states, impedance spectroscopy, topological perfect darkness

## Abstract

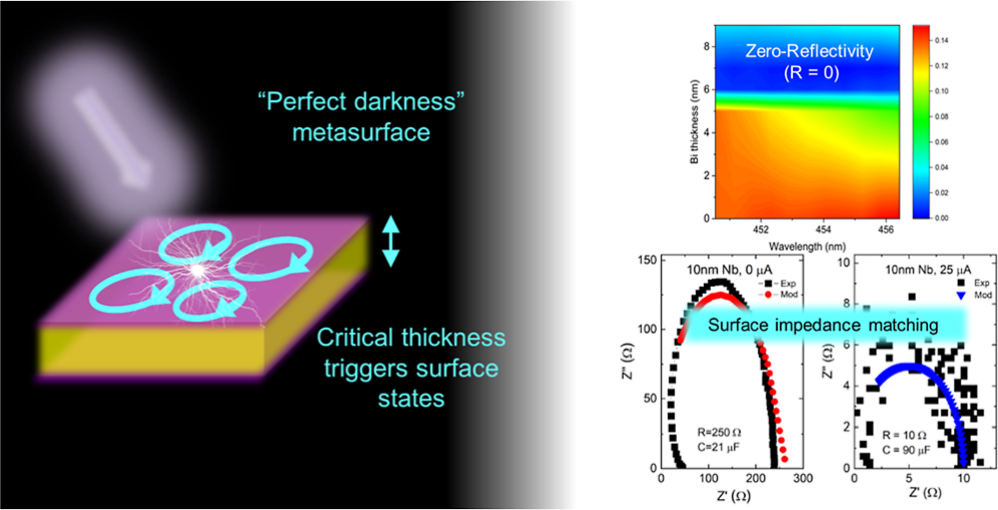

Metals, renowned for their high reflectivity, find extensive use in various technological applications, from mirrors to optical coatings in radars, telescopes, and mobile communications. However, their potential in antireflective coatings has remained largely untapped. In this study, we demonstrate that by applying an ultrathin metallic film onto an oxide layer, we can achieve a flawless optical surface with zero reflectivity. This phenomenon has been successfully observed across various metals, including Sn, Ag, Au, Pt, Bi, and Nb, showcasing its broad applicability. The underlying principle lies in the emergence of surface states, where the Rashba effect is strong, which give rise to the formation of Rashba metamaterial and metasurface (RMM) structures. Remarkably, these RMMs can be fine-tuned to act as high-resolution Veselago lenses. To illustrate, we achieved zero reflectivity with an RMM consisting of a 1 nm thick Sn metal film on a 1 nm Ge buffer, situated on a 60 nm Al_2_O_3_/Si substrate. Similar results were observed for other metals (Pt, Au, Ag, and Nb) and semimetals (Bi) by adjusting the film thickness to 2, 3, 5, 10, and 6 nm, respectively. The revelation of RMMs with zero reflectivity (*R* = 0) has tremendous potential to revolutionize optical device technologies, covering renewable energy, optoelectronics, and the telecommunications industry.

## Introduction

The search for materials, metamaterials, and metasurfaces with various important optical properties has expanded dramatically with the arrival of nanoscience. Optical coatings based on multilayer structures of dielectric films allowed us to obtain very low levels of light reflection.^1^ Despite recent successes, decreasing light reflection still remains one of the main obstacles in the construction of a perfect optical surface.

Current modern-day technologies use many methods to achieve antireflective coatings (see review^2^), frequently utilizing the inclusion of a quarter wavelength λ/4 interlayer/wave-plate to reduce the refractive index and control light transmission. However, even state-of-the-art antireflective coatings, achieving reflectivities as low as *R* ∼ 0.1% at certain specific wavelengths,^2,3^ have substantial limitations in terms of performance across a broad range of wavelengths. To remove light reflection over a broad-range spectrum, it is necessary to have an optical material with a graded refractive index, where its value gradually spans between two connecting interfaces (e.g., from air to substrate).^2,4,5^ There are many techniques for producing systems with a graded refractive index matching to ambient air, such as chemical vapor deposition, surface texture, and interference patterning.^6,7^ Recent examples of combining thin dielectric films with metallic substrates demonstrate how low reflection surfaces robust to the light’s angle of incidence can be achieved.^5^ Similarly, state-of-the-art dielectric metasurfaces and hybrid waveguides on a chip have sustained transmittance values of 98.5% at near-infrared wavelengths.^8,9^ Furthermore, the integration of graphene^10^ and its nanostructures^11,12^ has been shown to significantly affect the reflectivity of a surface. However, in all cases, reaching zero reflection over a broad wavelength range was still not possible.

**Figure 1:**
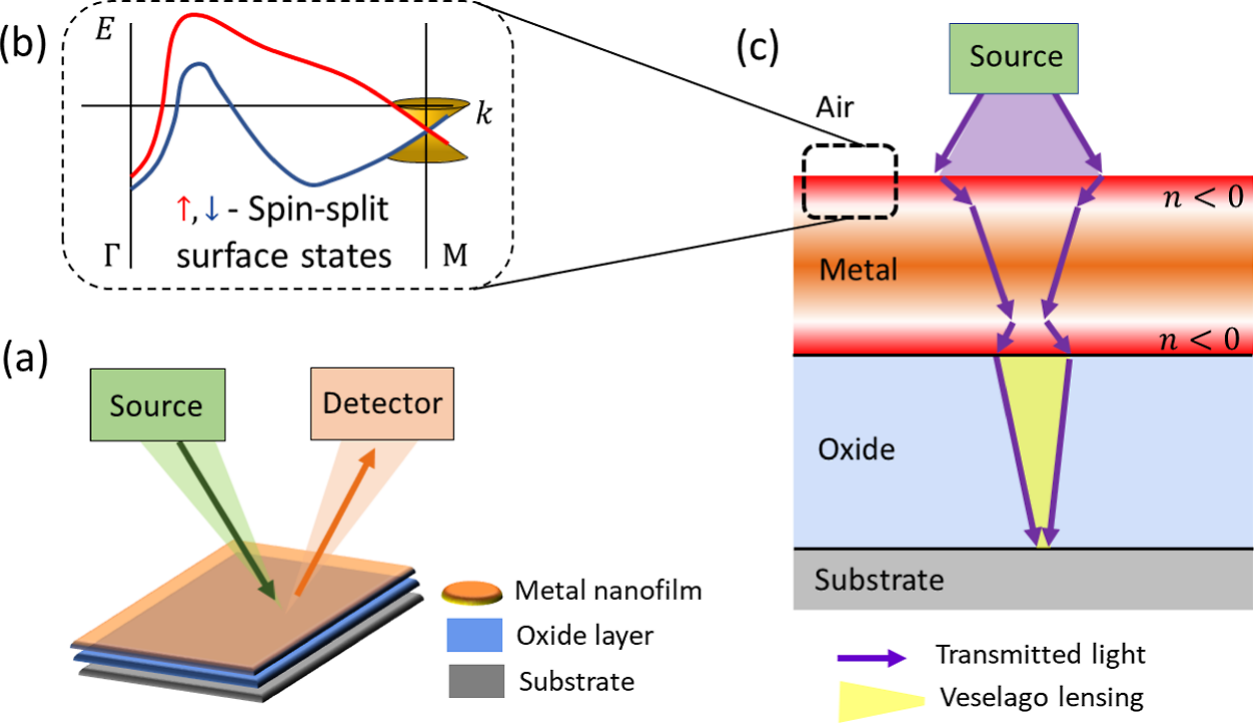
Schematic experimental setup for the reflectivity experiment. (a) The trilayer samples tested consist of a thick n-doped Si substrate (shown in gray); an insulating SiO_2_ or Al_2_O_3_ oxide layer with a thickness of 540 and 60 nm, respectively (shown in blue); and a metal film nm (shown in orange). The oxide layer between the metal film and Si substrate plays the role of the cavity in the Fabri–Perot resonator. The incident light is directed both perpendicular to the flat film surface and at some angles. The wavelength of the incident light is varied from near-ultraviolet (425 nm) to near-infrared (920 nm), covering all visible light frequencies. (b) Schematic example of the polarized SfSs that originate in the metal based on Bi nanofilms (showing a Dirac cone at the M point of the Brilloin Zone).^29^ (c) Schematic showing how zero reflectivity is achieved inside trilayers, due to the Veselago lensing effect arising near SfS regions with a negative refractive index (*n* < 0).

In the present work, we show how to obtain perfectly dark metasurfaces by optimizing trilayer structures composed of a substrate, an insulating oxide layer, and a metal nanofilm. The schematic diagram of the trilayer structures is shown in Figure 1. Typically, the thick substrate was made of n-doped Si, while the oxide layer was silicon oxide SiO_2_ or sapphire, Al_2_O_3_. The studied metal nanofilms included Sn, Ag, Au, Pt, Bi, and Nb metals with thicknesses ranging between 0.3 and 10 nm. We have found that the reflectivity vanishes (*R* = 0%) only at specific values of the metal nanofilm thickness, which depends on the type of material used. For Nb, Ag, and Pt metals, the effects seem to be anticorrelated with the element’s atomic number. The phenomenon occurs practically for all metals we have considered, in certain cases extending over a select frequency range. We show that a similar behavior can exist practically for all frequencies of light and extend to all metal films. The effect likely arises due to the presence of surface states (SfS) of metal nanofilm (Figure 1b) that play an increasingly significant role as the thickness of the film decreases.^13^

The SfS are effectively two-dimensional states in which the electrons are trapped near the surface. The perpendicular buckling deformation of the surface, which exists at any material interface due to strain, gives rise to an electric potential well, a Rashba field, confining the electrons. A Rashba field of this nature is tuned by deformation strain and may lead to strong spin–orbit coupling^14^ and topologically protected SfS. When the Rashba field is relatively weak, at low deformation strain, the SfS may behave as a 2D electron gas (2DEG), with localized or highly mobile electrons dictated by the two-dimensional electron density.^15,16^

Although the 2DEG has been well studied, there are many controversies surrounding the discoveries of metal–insulator transitions (MIT) in such systems.^17^ Specifically, while theories dictate that no metallic states can exist in 2DEG (in a zero magnetic field) due to Anderson localization,^15,18^ the interplay between the electron–electron interactions and disorder or deformation strain can trigger a competition between the kinetic and Coulomb energies in the electronic system. This can result in complex many-body ground states with competing orders^16,19,20^ and, in some cases, lead to an MIT and metallic behavior in 2DEG.^15^

Many new states of matter are predicted to arise in the proximity of an MIT in a homogeneous 2DEG. Most notably, these include the microemulsion phases,^21^ charge clusters described by a variational Jastrow-type many-body wave function,^16^ and the sliding quantum electron solids with a flat electronic spectrum.^22^ Charge cluster effects have been recently observed in ZnO-based two-dimensional electron systems^23^ and low-dimensional semiconductors.^24−27^ It is generally accepted that the MIT transition in a 2DEG is tuned by an electron density variation.^28^

Our studies of the trilayers demonstrate that we can trigger an MIT transition in the SfS by varying the thickness of the metal nanofilm and thus changing its electron density. At a critical film thickness, the metal nanofilm transforms into two metallic 2D SfS layers that sandwich the bulk, which is insulating due to size quantization (Figure 1c). The metallic SfS creates narrow regions of negative refractive index *n* < 1 at the metal nanofilm/oxide interfaces. Consequently, due to strain-induced perpendicular buckling at the interfaces, the trilayer becomes an alternating structure of nanoscale metal and insulator multilayers with strong local Rashba spin–orbit coupling—a Rashba metamaterial and metasurface (RMM). Such an alternation of metal and insulating layers will dramatically change the dielectric tensor of the system, causing a transformation in the permittivity response, analogous to those found in hyperbolic metamaterials.^30−32^ We show that all trilayers can be tuned toward RMMs, where the effective refractive index matches that of air and the reflectivity is zero, by varying the thickness of the metal nanofilm. RMMs may also be considered as flat Veselago lenses with extremely high resolution due to the nanometer size of the *n* < 1 SfS regions at the metal nanofilm/oxide interfaces. The Veselago lensing may focus incident light further into the trilayer structure without reflection losses, making RMMs a unique example of systems that demonstrate perfect antireflection.

Our discovery of the zero reflectivity phenomenon based on RMMs is universal and offers a diverse playground for device design and fabrication. The technology developed around this new effect has significant potential for a variety of applications ranging from solar cells to optical sensors and quantum technologies, progressively impacting the energy, space, and communications sectors.

## Results

### Antireflective Properties

The Sn trilayers were composed of an Sn nanofilm deposited onto a Ge layer grown on top of 60 nm Al_2_O_3_ oxide/n-doped Si substrate. The Ge layer acted as a wetting buffer, ensuring a more homogeneous spread of Sn around the Al_2_O_3_ surface. The best optical response was achieved when the thicknesses of Ge and Sn were equal.

**Figure 2:**
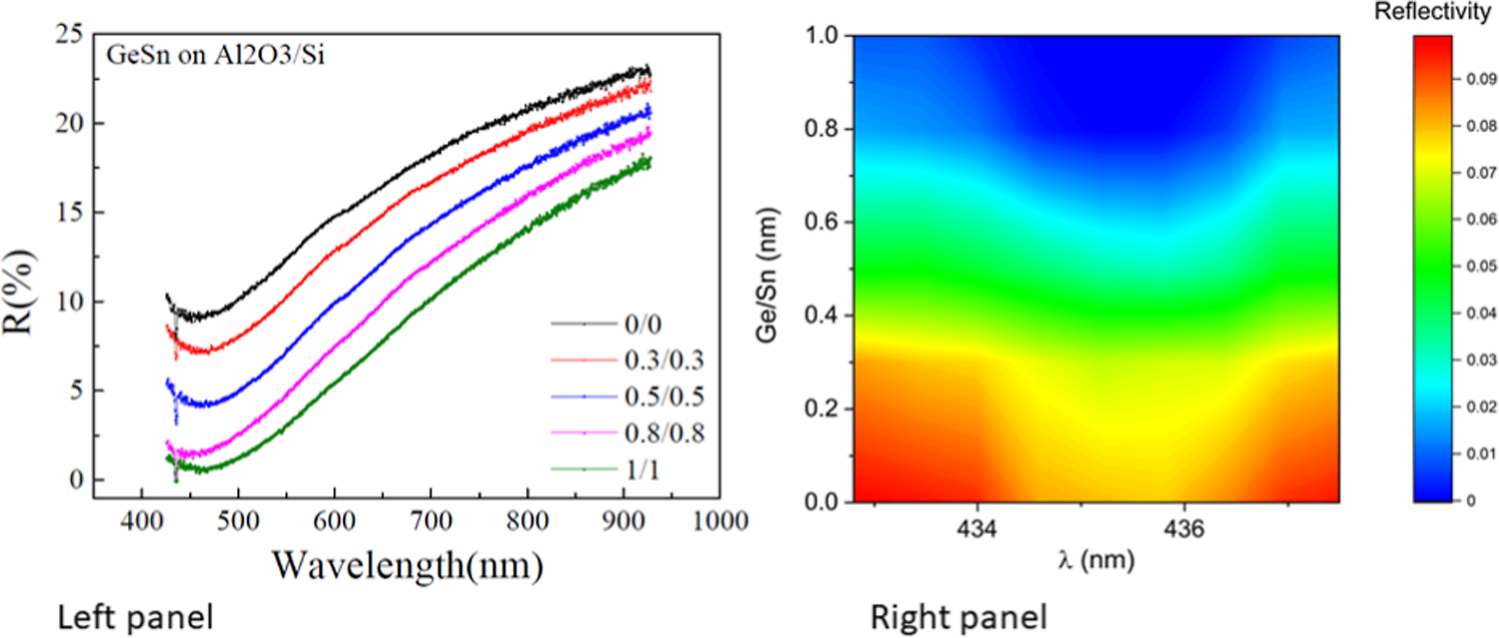
Left panel: Reflectivity for Ge/Sn trilayers over the visible light frequency range. The wavelengths are given in nm. The studied Ge/Sn film thicknesses were 0/0 nm (black), 0.3/0.3 nm (red), 0.5/0.5 nm (blue), 0.8/0.8 nm (purple), and 1.0/1.0 nm (green), respectively. The broad minimum (in all curves) around 450–460 nm corresponds to the λ/4-wavelength destructive interference in the Fabry–Perot resonator made from 60 nm Al_2_O_3_ oxide. At 430–440 nm, there is a further significant decrease in reflectivity. Right panel: Dependence of the reflectivity on incident wavelength and the thickness of the Ge/Sn nanofilm in the region of 430–440 nm. Zero reflectivity regions are colored blue and occur for Ge/Sn films with thicknesses of 0.8 and 1.0 nm.

Multiple samples with Ge/Sn thicknesses 0.3/0.3, 0.5/0.5, 0.8/0.8 nm, and 1.0/1.0 nm have been fabricated and their reflectivity spectra studied Figure 2 (left panel). All reflectivity spectra contain a broad minimum around the 450–460 nm wavelength. Interestingly, as the thickness of the Ge/Sn films increases, the reflectivity decreases for all studied wavelengths. However, the position of the broad reflectivity minimum remains the same across all samples. That means that the thickness of the Ge/Sn nanofilm has no effect on the length of the optical path inside the trilayers, which is in strong contrast to conventional dielectrics. For comparison, when the Ge/Sn thicknesses are greater than 1 nm, the reflectivity begins to increase again (see SI Figure 1). In addition, all samples show a sharp drop in the reflectivity to beyond the measuring resolution of the instrument (≪ 0.01%) at 430–440 nm wavelengths. This behavior occurs over the same wavelength range, irrespective of the Ge/Sn thickness [see Figure 2 (right panel)]. Notably, at the Ge/Sn nanofilm thicknesses between 0.8 and 1 nm, the Al_2_O_3_ oxide transforms into a perfectly dark metasurface with zero reflectivity (*R* = 0%) over a narrow range of wavelengths [see Figure 2 (right panel in blue)].

This behavior may be explained by considering the 60 nm thick Al_2_O_3_ oxide as a Fabry–Perot resonator (FPR), sandwiched between the Ge/Sn nanofilm and the Si substrate. Inside the FPR resonator, the reflectivity minimum arises due to destructive interference when the thickness of the FPR is equal to 1/4 of the wavelength of incident light. Here, the destructive interferences occur when the waves reflected from the top of the Ge/Sn metal nanofilm and from the bottom of the Al_2_O_3_ oxide are out of phase, with a phase shift of π. If the amplitudes of the two interfering waves are equal, then there is complete wave annihilation, giving rise to zero reflectivity. For that to happen, the quality of the FPR must be very high with virtually no losses. Our results clearly demonstrate that coating the effective Al_2_O_3_ oxide FPR with a Ge/Sn nanofilm at a critical thickness between 0.8 and 1 nm significantly enhances its quality factor, transforming it into a perfect antireflector, which surpasses the performances of many existing antireflective surface coatings^33−37^.

However, the exact effect of the metal nanofilm is rather subtle. Bulk metals normally have good optical conductivity, which is normally expressed by a large extinction coefficient. When a metal film thickness approaches the nanometer scale, the bulk optical conductivity vanishes. The metal nanofilm becomes effectively insulating unless the metallic SfSs are taken into account. Such SfSs provide remnant metallicity, signified by a Dirac spectrum as in graphene or other topologically protected states. Thus, in metal nanofilms, the extinction coefficient and optical conductivity are defined by metallic SfSs, not by the insulating bulk. By changing the Sn/Ge nanofilm thickness, layer by layer, we transform the Sn/Ge trilayers into an RMM and significantly modify the optical constants to achieve zero reflectivity.

Notably, the SfSs in Ge/Sn trilayers are unique because the presence of the buffer Ge layer stabilizes a preferential α—Sn or Gray Tin phase at the boundary. α—Sn is known to be a strong topological insulator, and its proximity to Ge leads to the appearance of a metallic phase with a linear electronic spectrum, a Dirac semimetal, due to a mutually inverted band structure. (The valence band of Ge is transformed to the same irreducible representation of the symmetry group as the conduction band of Sn.) The Dirac semimetal based on the Ge/α—Sn interface has been predicted theoretically^38^ and recently realized experimentally.^39^ Dirac semimetals show large mobilities of the conduction carriers, ∼30,000 cm^2^ V^–1^ s^–1^, indicative of the presence of massless Dirac electrons, which have symmetry protection from conventional (except magnetic) scattering mechanisms. Thus, the interaction between the SfSs associated with the different sides of the Sn nanofilm in the Ge/Sn trilayers may lead to the formation of very nontrivial electronic characteristics. For example, it was shown recently that covalently bonded Ge and Sn monolayers give rise to a new class of 2D materials, with metallic-like signatures in the optical conductivity at lower energies.^40^

**Figure 3:**
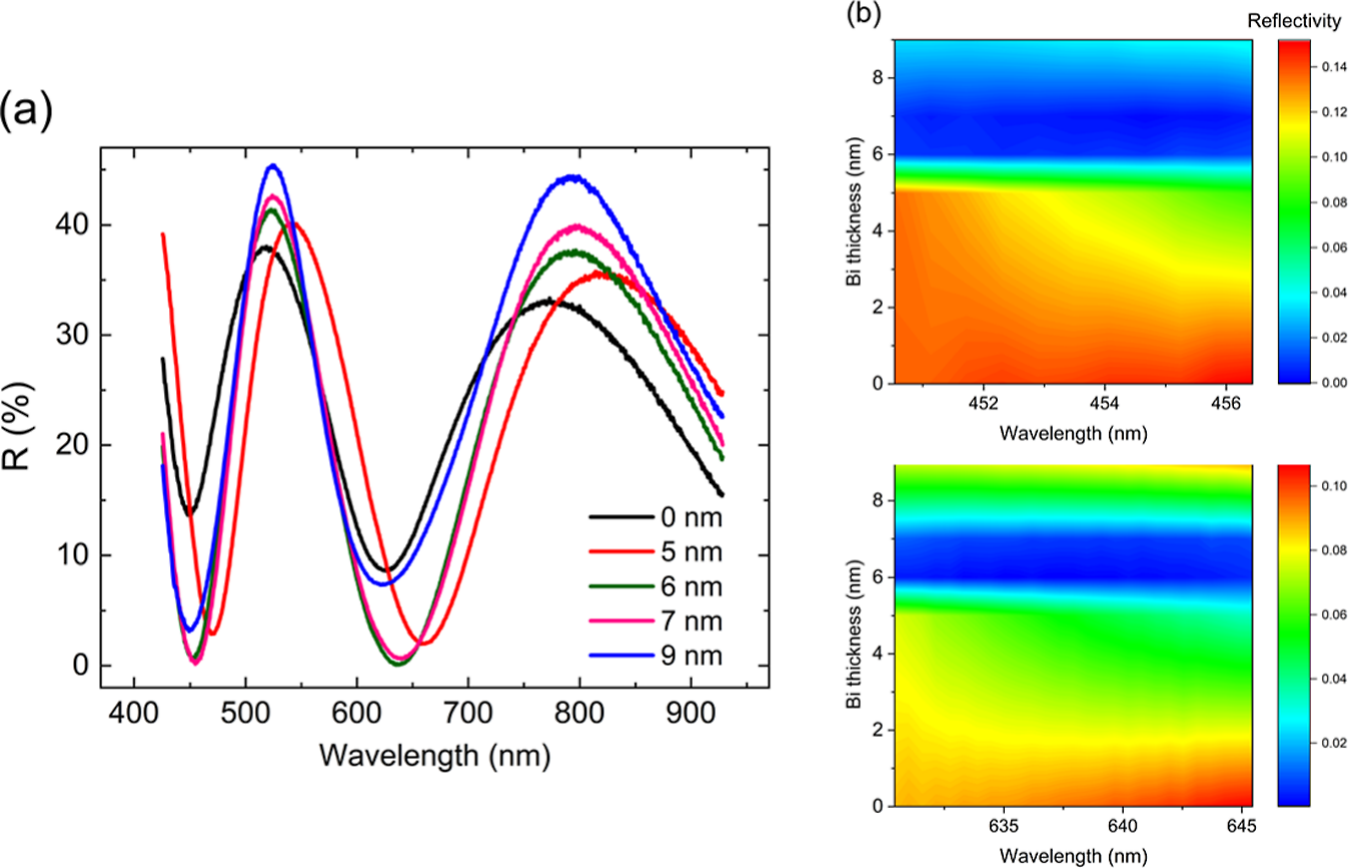
Dependence of the reflectivity on the light wavelength for Bi nanofilms. (a) The Bi nanofilms were grown on a 540 nm thick SiO_2_ oxide layer. The Bi nanofilms had thicknesses of 5 nm (red), 6 nm (green), 7 nm (pink), and 9 nm (blue). Two zero reflectivity minima are observed at nanofilm thicknesses of 6 nm (green curve) and 7 nm (pink curve). The zero reflectivity minima occur at light wavelengths 642 nm and 455 nm, respectively. (b) Color phase diagram depicting the zero reflectivity regions for Bi nanofilms as a function of film thickness (nm) and light wavelength (nm). The zero reflectivity is shown in blue.

**Figure 4:**
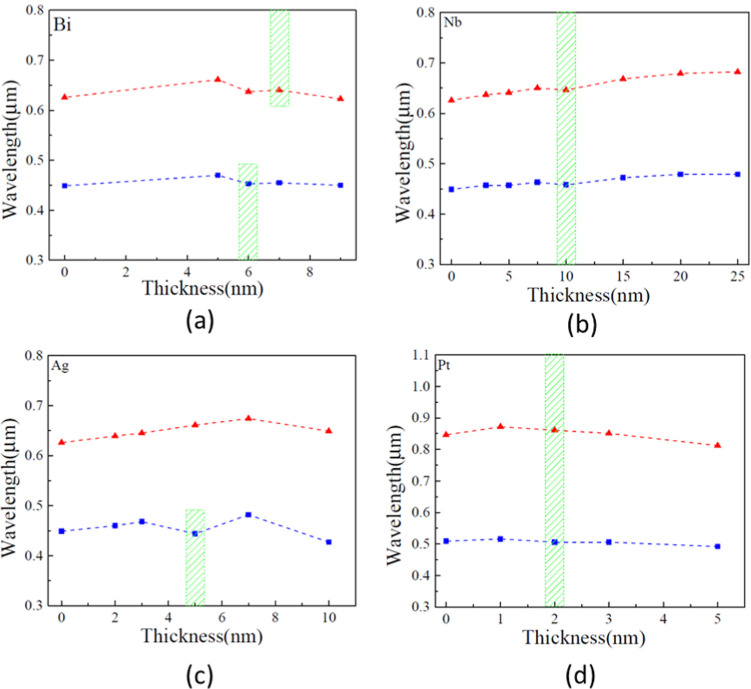
Dependence of the wavelength at which the minimum of reflectivity is observed on the thickness of the metal nanofilms in the studied trilayers. Blue and red points correspond to the positions of the low-wavelength and high-wavelength minima, respectively. The Nb, Bi, and Ag films have been deposited on 540 nm of SiO_2_ oxide, while the Pt film was grown on 430 nm of SiO_2_ oxide. The film thickness at which zero reflectivity is observed is shown as a green shaded area. (a) Bi films show zero reflectivity at 6 and 7 nm, (b) Nb films show zero reflectivity at 10 nm, (c) Ag films show zero reflectivity at 5 nm, and (d) Pt films show zero reflectivity at 2 nm of thickness. All graphs demonstrate that the wavelengths at which reflectivity minima occur are fairly insensitive to the thickness of the metal nanofilm.

To underline the generality of our approach, the zero reflectivity effect has been seen in many other trilayer structures. Trilayer samples with Bi nanofilms also demonstrate zero reflectivity at nanofilm thicknesses of 6–7 nm. The Bi systems were deposited onto a 540 nm SiO_2_ oxide layer grown on an n-doped Si substrate. The reflectivity spectra for four Bi nanofilms with thicknesses of 5, 6, 7, and 9 nm are shown (Figure 3a). The zero reflectivity minima are confirmed near incident light wavelengths of 642 and 455 nm for the 6 and 7 nm thick Bi film, respectively. The zero reflectivity behavior is clearly illustrated in Figure 3b (blue regions). It is evident that the position of the reflectivity minima does not depend significantly on the thickness of the Bi nanofilm (Figures 3a and 4a), while the reflectivity changes drastically. Similar to the Sn/Ge trilayers, it is natural to conclude that the Bi nanofilms do not contribute to the length of the optical path. The light interference occurs mostly inside the SiO_2_ layer sandwiched by Bi film and n-doped Si from both sides, which behaves as the effective FPR. As before, the presence of the Bi nanofilms on the surface of the oxide significantly improves its quality factor, leading to the two observed zero reflectivity minima (Figure 3b) when the Bi thickness is critically tuned to transform the system into an RMM.

To investigate our premise that the conversion of the trilayers in RMMs may be related to an MIT transition in the 2DEG confined in SfSs, we focus on the Bi systems. Early theoretical work^41,42^ has predicted an MIT to occur in Bi films at a thickness of 40 nm, which has been extensively confirmed by experiments.^43,44^ Conversely, recent ARPES experiments have found metallicity in Bi metal films with thicknesses below 20 nm.^45^ This was explained through the existence of SfSs with nontrivial electronic topology.^46^ For topologically protected SfSs, the Fermi surface shows strong spin–orbit splitting and coupling due to the breaking of the spatial inversion symmetry.^14,46^ Thus, the SfSs give rise to spin-polarized surface bands,^47,48^ where the carrier density is nearly 2 orders of magnitude higher than in the bulk of the film. Consequently, the resistance of the Bi(111) films remains independent of its thickness in the 4–30 nm range, plateaus at a value of 8.8 kΩ, and demonstrates classical magnetoresistance behavior.^47^ For this reason, the SfSs provide the dominant channel for charge transport in Bi nanofilms. The effect arises when the Bi thicknesses decrease to 7–9 nm. This is in good agreement with our experimental observations of perfect antireflection in the Bi trilayers, indirectly corroborating the scenario that an MIT in the SfSs and the zero reflectivity phenomenon may be related. Additionally, Bi lies very close to a phase boundary between a strong topological insulator and higher-order topological phases^49^ which could be stabilized by the presence of any grains or grain edges.

The nature of the SfS in Bi nanofilms has been further clarified in recent, pioneering high-resolution ARPES work that revealed a new, unexpected mechanism for the MIT in Bi.^29^ It was shown that as the thickness of the Bi(111) films decreases to the nanoscale, the topological deformation of the bulk bands associated with the lower energy states, due to quantum confinement, gives rise to a striking degeneracy of the two SfS bands. Thus, the lowest-energy quantized bulk state transforms into a surface-localized state, establishing a conducting channel on the surface. The effective shift of the band edge makes the interior of the Bi film insulating, as shown in previous experiments.^47,48^ It is possible that this would be accompanied by a charge redistribution between the bulk and the surface, indicating the importance of the Coulomb interaction in the formation of the SfSs.

Our results illustrate that the conversion of trilayers into RMMs is completely universal and can be tuned by changing the thickness of the metal nanofilm. The proposed method for achieving metamaterials and metasurfaces can be generalized to a new class of size effects in any dimension. Specifically, as the system size is reduced to the nanoscale, the formation of the 1D and 2D edge state conduction channels due to strong Coulomb forces may have a dominant effect on the optical conductivity. The metallicity in these conducting channels, resulting from a possible nontrivial MIT as in 2DEG,^15−19^ can drastically change all optoelectronic properties. Present work demonstrates that the RMMs tuned from the trilayers’ manifest zero reflectivity and associated gradual matching of refractive index behaviors. Using the simple and robust proposed strategy, diverse trilayers can be designed into RMMs for a wide range of applications.

### Modeling Antireflection in Metallic Nanofilm/Oxide Systems

We have illustrated experimentally that changes in the metallic nanofilm thickness, layer by layer, may tune its optical constant significantly and modify antireflection properties to a desired effect, including zero reflectivity. In the studied metallic trilayer structures, the metal nanofilm contains two SfS layers, one at the vacuum and one at the oxide interface. The SfS may be considered effectively as a 2DEG and thus may exhibit similar metallic character and MIT when the electron density decreases. The latter can be changed with the number of metallic monolayers forming the film, where the electron density increases with an increasing film thickness. The MIT transformation in SfSs would influence substantially the optical responses of the metal nanofilm and the underlying oxide. Specifically, for the optical case of zero reflectivity, this would be expressed as a negative permittivity, a negative refractive index (Figure 1c), and a large extinction coefficient (Table 1)—contributing to the Veselago lensing effects in these systems. The processes described here is completely different from those found in dielectric materials.

**Table 1:** Optical Constants for Different Metal Nano-Films Used in Trilayer Systems with Zero Reflectivity

metal	permittivity ϵ_1_	refractive index *N*_1_	thickness *d*_1_ (nm)	wavelength λ (nm)
Sn	14.86–34.6*i*	5.12–3.38*i*	1	435
Sn	19.9–33.1*i*	5.4–3.6*i*	1	450
Bi	–10.1–0.8*i*	0.13–3.18*i*	7	453
Bi	–9.1–3.9*i*	0.6–3.08*i*	6	637
Nb	–5.1–1.45*i*	0.32–2.28*i*	10	459
Nb	–3.9–2.7*i*	0.69–2.02*i*	10	646
Ag	–3.64–7.276*i*	1.5–2.4*i*	5	659
Pt	–26.9–11.28*i*	1.06–5.3*i*	2	505
Pt	–1.9–26.98*i*	3.54–3.8*i*	2	843

Would it be possible to describe the effects arising from SfSs using Fresnel theory, which assumes that the medium is homogeneous and isotropic, containing flat interfaces? The SfS usually creates an electron density profile through the surface cross section. For all studied systems (see, Figures 2–4), the SfSs are sandwiching the insulating bulk part of the metal nanofilm, where the respective thickness of the SfS and the bulk are comparable. Thus, in order to obtain the optical constant of the metal nanofilm, we have to calculate its optical conductivity, which includes charge transport through the two SfS layers and the insulating bulk. This is a formidable task. To simplify, we may effectively assume that the refractive and extinction coefficients of the nanofilm are known and apply the Fresnel theory directly to fit the experimental data. In this case, the optical constants of the nanofilm may be considered as fitting parameters.

In our approach, the incident light is assumed to be a plane wave, which is appropriate because any incident light field can be decomposed into plane waves and its polarizations. We model trilayer structures, consisting of a metal nanofilm (∼1–10 nm thick) and an oxide (SiO_2_ or Al_2_O_3_) deposited on the n-doped Si surface. Our goal is to determine whether coating the oxide with a metal nanofilm will significantly change the effective refractive index of the trilayer system and possibly even make it match the refractive index of air. In this way, zero reflectivity may be achieved for the entire trilayer. By using the Fresnel theory combined with the transfer matrix method, one may immediately find a complex effective optical constant, *N*_eff_, and reflectivity of the trilayer system. Naturally, the effective reflectivity, *R*_eff_, depends on the multilayer structure, the thicknesses of the individual layers, *d*_*i*_, and their optical constants, *N*_*i*_, where the index *i* runs through the number of layers comprising the multilayer. For zero reflectivity, we must have the effective optical constant of the system, *N*_eff_, matching the optical constant of air, *N*_eff_ = 1 + *i*0. Because the metal nanofilms are extremely thin, we can define a small phase parameter, δ_1_ = 2π*N*_1_*d*_1_/λ ≪ 1, where *N*_1_(*d*_1_) is the optical constant (thickness) of the metal film. By making an expansion of the effective trilayer optical constant *N*_eff_ in terms of this small parameter δ_1_, a simplified expression for *N*_eff_ is obtained that can be analyzed analytically. The exact details of the theory are presented in the Supporting Information file. The effective optical constant for the described trilayer setup will have the form
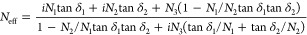
1where *N*_1_, *N*_2_, and *N*_3_ are the optical constants of the metal nanofilm, the oxide layer, and the Si substrate, respectively. δ_1_ = 2π*N*_1_*d*_1_/λ and δ_2_ = 2π*N*_2_*d*_2_/λ are the phase parameters associated with the metal nanofilm and the oxide and *d*_1_ and *d*_2_ are their respective thicknesses. Here, for simplicity, only normal incidence electromagnetic waves are considered. *N*_eff_ = *n*_eff_ + *ik*_eff_; the effective reflectivity is calculated with the aid of the standard equation, . The dependence of the metal nanofilm thickness *d*_1_ = λδ_1_/2π*N*_1_ on the effective refractive index *N*_eff_ can be found directly in eq 1. Consequently, measuring the effective optical constant of the system *N*_eff_ (or its reflectivity and the light absorption) will provide the dependence of the refractive index and extinction coefficient of the metal film, *N*_1_, on the film thickness *d*_1_.

The proposed framework is applied to determine the dielectric constant (or permittivity) ϵ_1_(*d*_1_,λ) = ϵ_1_^′^(*d*_1_,λ)+*i*ϵ_1_^″^(*d*_1_,λ) and the optical coefficients of the metal nanofilm, *N*_1_ = *n*_1_ + *ik*_1_, at minima where zero reflectivity is established experimentally in the studied films of Bi, Nb, Sn, Ag, and Pt. The zero reflectivity conditions are summarized in Figure 4 and Table 1. In particular, Bi nanofilms have shown two zero reflectivity regions at λ = 637 nm and λ = 453 nm in films with thicknesses *d*_1_ = 6 nm and *d*_1_ = 7 nm, respectively (Figure 3, Table 1). Here we have found that the dielectric function of the Binanofilm at the first zero reflectivity minimum in Bi (*d*_1_ = 6 nm and λ = 637 nm) is equal to ϵ_1_(6, 637) = −9.1–3.9*i*. By taking a square root of the ϵ_1_(6, 637), the film’s optical constant is obtained: *N*_1_(6, 637) = 0.6–3.08*i*. Similarly, for the second short wavelength minimum (*d*_1_ = 7 nm and λ = 453 nm), the dielectric constant is ϵ_1_(7, 453) = −10.1–0.8*i*, giving the film’s optical constant as *N*_1_(7, 453) = 0.13–3.18*i*. By comparing the *N*_1_ across the two minima, it becomes clear that the extinction coefficient *k*_1_ changes only slightly, while the refractive index *n*_1_ declines substantially. The results are consistent with our conjecture that the zero reflectivity observed here may be connected to the formation of charged clusters in 2DEG, which may be a precursor of a nontrivial MIT arising there when the electron density decreases.^15−19^ In this case, the charged inhomogeneities or clusters^16^ may be responsible for an increase in the imaginary part of dielectric function, ϵ″(*d*_1_, λ), associated with light absorption. Notably, the imaginary parts of the dielectric function are substantially larger for the Bi nanofilms with a thickness of 6 nm compared to those with a thickness of 7 nm; ϵ_1_^″^(6637) = -3.9 ≫ ϵ_1_^″^(7453) = −0.8. One may postulate that the 6 nm thick Bi contains many more charged clusters compared with 7 nm thick Bi, signaling a possible MIT transition tuned by the thickness of the Bi layers. Also, as a result of the possible MIT transformations in the SfSs, the extinction coefficient is significantly larger than the refractive index, which is smaller than 1 for both these Bi nanofilms (see Table 1).

Nanofilms of Nb, Ag, and Pt demonstrate similar behavior to Bi. In all cases, negative permittivities ϵ_1_ were obtained at the zero reflectivity minima (see Table 1). Nb films show two zero reflectivity regions at λ = 459 nm and λ = 646 nm, both at film thickness *d*_1_ = 10 nm (Figure 4, Table 1). For the short-wavelength minimum (*d*_1_ = 10 nm and λ = 459 nm), the permittivity is ϵ_1_(10, 459) = −5.11278–1.44812*i* giving optical constant as *N*_1_(10, 459) = 0.317–2.28*i*. For the long-wavelength minimum (*d*_1_ = 10 nm and λ = 646 nm), the permittivity is ϵ_1_(10, 646) = −3.9–2.7*i*, giving the film’s optical constant as *N*_1_(10, 646) = 0.69–2.02*i*. Similar to Bi films, in Nb, the extinction coefficient changes only slightly and dominates the optical response.

Ag nanofilms showed only one zero reflectivity minimum at λ = 659 nm and film thickness *d*_1_ = 5 nm (Figure 4, Table 1). The permittivity was found to be ϵ_1_(5, 659) = −3.6398–7.26976*i*, giving an optical constant of *N*_1_(5, 659) = 1.5–2.4*i*. The strongly negative permittivity and dominant extinction coefficient values again indicate the metallic nature of the optical response. Although, Pt nanofilms were grown on a slightly thinner 430 nm SiO_2_ oxide layer, the general trend remains unchanged. Pt films showed two zero reflectivity minima at λ = 505 nm and λ = 843 nm, both at film thickness *d*_1_ = 2 nm (Figure 4, Table 1). At the short-wavelength minimum (*d*_1_ = 2 nm and λ = 505 nm), the permittivity and the optical constant are ϵ_1_(2, 505) = −26.934–11.2766*i* and *N*_1_(2, 505) = 1.06–5.3*i*, respectively. At the long-wavelength minimum (*d*_1_ = 2 nm and λ = 843 nm), the following permittivity ϵ_1_(2, 843) = −1.92013–26.9774*i* and optical constant *N*_1_(2, 843) = 3.54–3.8*i* values are obtained.

The hybrid Sn trilayers have the structure of 1 nm Sn/1 nm Ge/60 nm Al_2_O_3_/Si. The sample shows a near zero reflectivity (*R* % ∼ 0.5%) region at a wavelength of λ = 450 nm and complete zero reflectivity at a wavelength of λ = 435 nm (Figure 2). The permittivity at the long-wavelength broad minimum is ϵ_1_(1, 450) = 19.9–33.1*i*, while the film’s optical constant is *N*_1_(1, 450) = 5.4–3.6*i*. The permittivity at the short-wavelength zero reflectivity minimum is ϵ_1_(1, 435) = 14.86–34.6*i*, giving the optical constant as *N*_1_(1, 435) = 5.12–3.38*i*.

**Figure 5:**
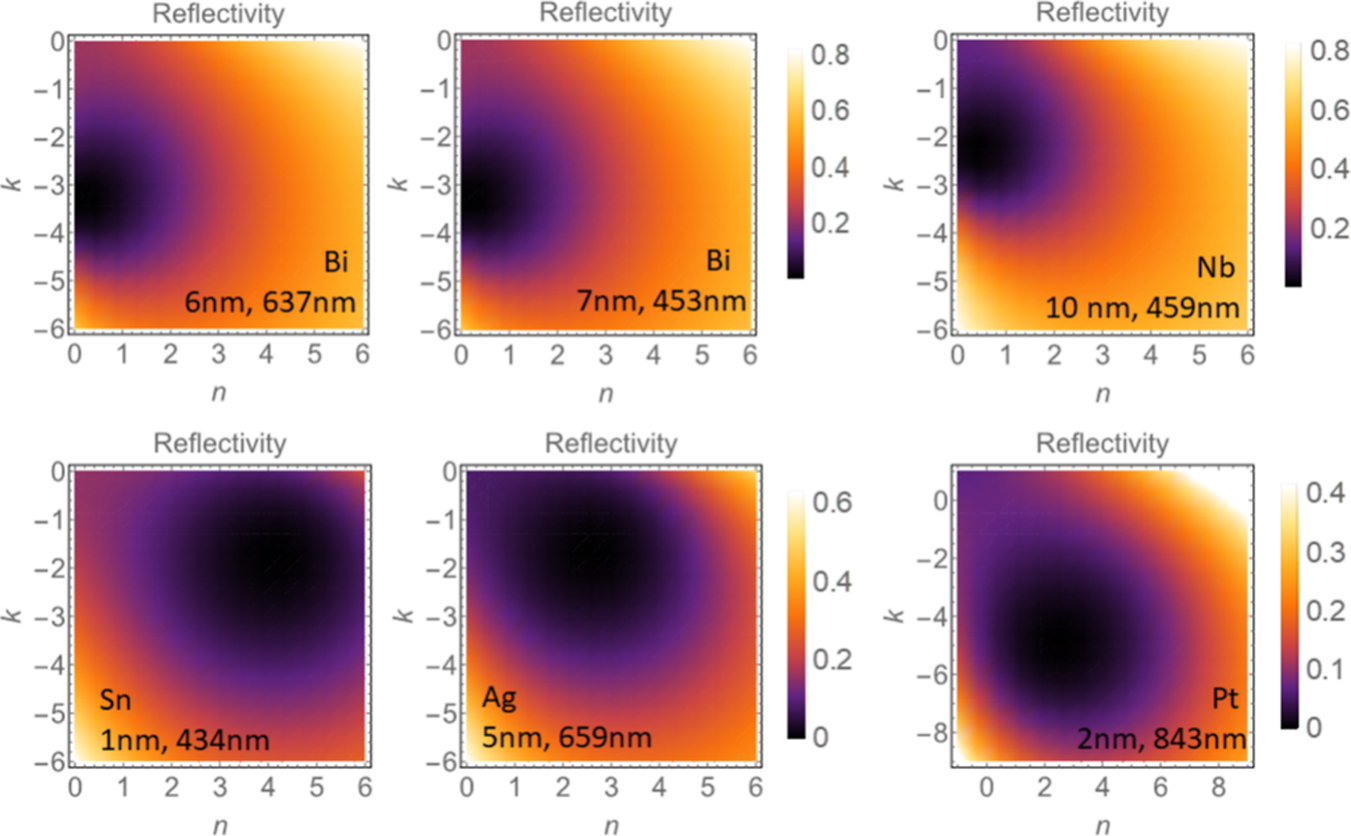
Dependence of the reflectivity on optical parameters *n* and *k* calculated at film thickness *d*_1_ and light wavelength λ corresponding to the zero reflectivity position. Black/dark purple regions correspond to zero reflectivity. The considered metal nanofilms are Bi *d*_1_ = 6 nm, λ = 637 nm and *d*_1_ = 7 nm, λ = 453 nm; Nb *d*_1_ = 10 nm, λ = 459 nm; Sn *d*_1_ = 1 nm, λ = 434 nm; Ag *d*_1_ = 5 nm, λ = 659 nm and Pt *d*_1_ = 2 nm, λ = 843 nm. Note that in all these cases, the minima position is dispersed and the zero reflectivity area associated with dark spots of these plots is very broad, i.e., it depends weakly on the value of the refractive index and the extinction coefficient.

To complete our understanding of the trilayer systems, we have used our phenomenological approach based on the Fresnel theory to calculate the reflectivity profile as a function of the optical constants (*n* and *k*) around the zero reflectivity minima. The reflectivity was obtained by using eq 1 while constraining the value of *N*_1_(*d*_1_) in the trilayer samples. For all calculated samples, we have fixed the nanofilm thickness *d*_1_ and the light wavelength λ to the values that correspond to zero reflectivity in the experimental measurements. No fitting parameters were used. The calculated results for Bi, Nb, Sn, Ag, and Pt nanofilms are shown in Figure 5 and illustrate the reflectivity profiles of the different films as a function of the refraction and extinction coefficients *N*_1_ = *n*_1_ + *ik*_1_. The zero reflectivity regions are indicated in dark purple/black. Note that all of these zero reflectivity spots, see the plots in Figure 5 are very broad, which indicates that the zero reflectivity devices can be made by a variety of ways. The theoretical calculations agree extremely well with the experimental observations for all films. All optical constants *N*_1_ that were obtained by fitting the experimental reflectivity results at the zero reflectivity minima (see Figures 2 and 3, and Table 1) fall firmly within the corresponding theoretically calculated zero reflectivity profiles for each film (see Figure 5). The general consistency and striking agreement between the theoretical predictions and the experimental data further support our interpretation that the optical response and reflectivity phenomena in metallic nanofilms is dominated by the SfSs and their properties.

### Raman Spectroscopy and Surface Phonon Modes

**Figure 6:**
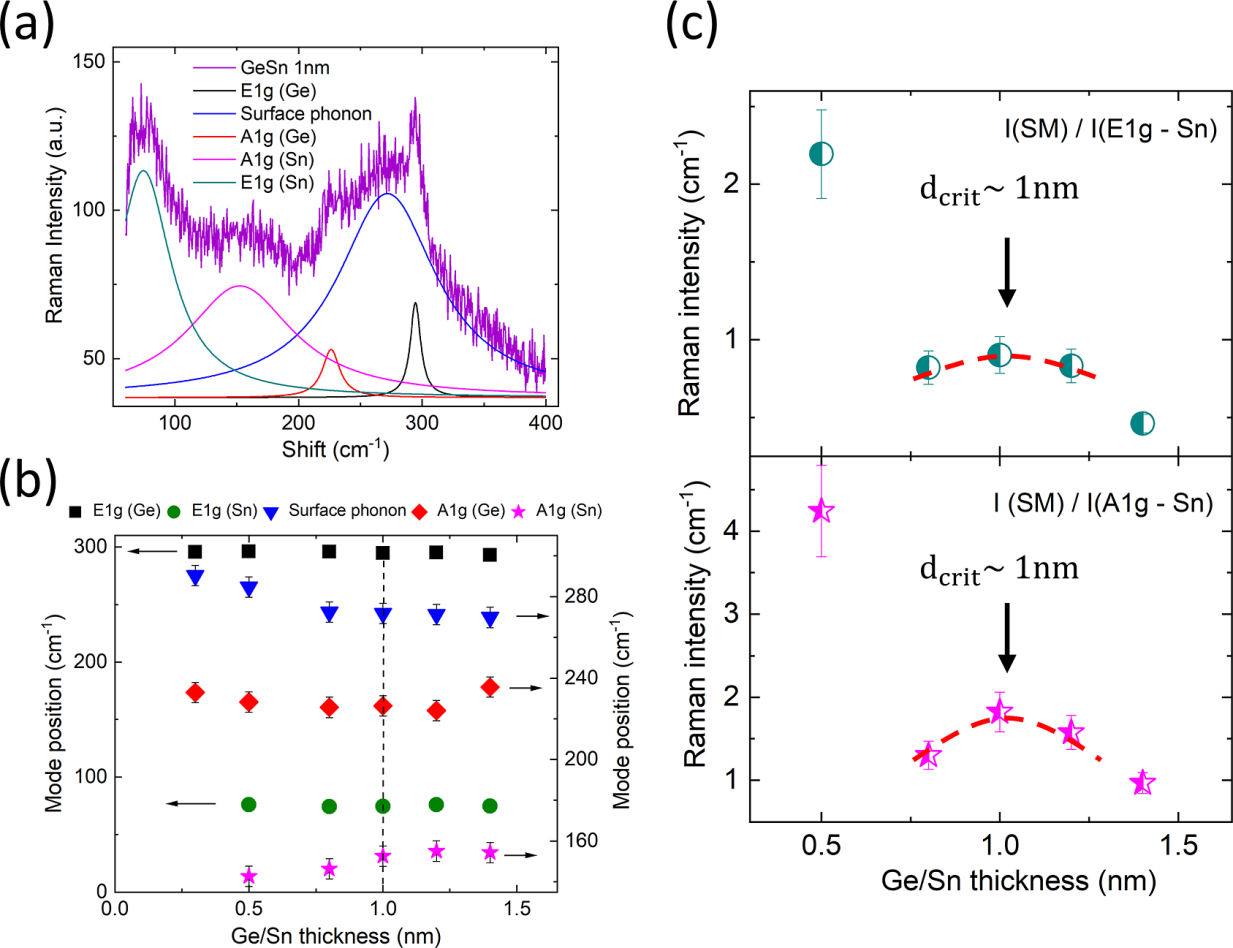
Raman spectroscopy results for the hybrid Sn trilayer samples. (a) Raman spectrum for the Ge/Sn trilayer, with individual-layer critical thicknesses; *d*_crit_ = 1 nm—where zero-reflectivity was observed. E1g and A1g modes for Ge and Sn are indicated in black, green, red, and pink, respectively. The surface mode is shown as a broad blue peak. (b) Dependence of the identified E1g, A1g, and surface modes as a function of Ge/Sn thickness. E1g mode for Ge (black squares), E1g mode for Sn (green circles), A1g mode for Ge (red diamonds), A1g mode for Sn (pink stars), and the surface mode (blue triangles) are shown. (c) Dependence of the intensity ratio for the surface mode *I*(SM) and the corresponding *I*(E1g) (top) and *I*(A1g) (bottom) modes of Sn. Critical thickness *d*_crit_ = 1 nm is indicated.

The inelastic scattering of light in Raman spectroscopy is a sensitive probe for studying surface phonons and other surface effects.^50,51^ The energy transfer between light and the phonons in the lattice can be substantially different between the bulk and the surface.^50^ While in thick bulk films, the surface Raman spectroscopy effects are negligible, they become significant when the film thickness decreases to the nanometer scale. In particular, it was demonstrated that the Raman intensity of the surface phonon modes increases substantially as the film layer thickness decreases to several monolayers.^51,52^ Thus, hybrid Sn trilayers were probed with Raman spectroscopy to observe the presence of SfSs and how it may relate to the optical properties,; see Figure 6. The thickness of the Ge/Sn in the hybrid Sn trilayer samples was varied between 0.3 and 1.4 nm, and all Raman spectra displayed several characteristic vibrations below 400 cm^–1^. As an example, the Raman spectra of the hybrid Sn trilayer with the critical thickness *d*_crit_ = 1 nm is shown in (Figure 6a). The critical Sn film thickness of 1 nm corresponds exactly to the conditions where zero reflectivity is observed in Sn nanofilms, *d*_1_ = *d*_crit_ = 1 nm. The Raman peaks observed at 295 and 233 cm^–1^ correspond to the longitudinal E1g and the transverse A1g vibrational modes of the Ge atoms, respectively.^53^ The Ge vibrations can be fitted by a narrow Lorentzian (fwhm ∼15 cm^–1^), indicating good crystallinity of the layers. The frequency of the Ge modes is shifted to lower energies compared to those found in the Ge bulk material, which may be attributed to the proximity and strain coming from the Sn layer.^54,55^ The Ge E1g and A1g modes are marked in black and red, respectively (see Figure 6a). The broad (fwhm ∼100 cm^–1^) blue peak found at 280 cm^–1^ near the Ge E1g maximum is attributed to surface phonon modes (SM) that are related to the SfSs.^51^ The Sn longitudinal and tranverse E1g and A1g modes are observed at the expected frequencies of 80 and 140 cm^–1^, respectively^56,57^ (shown in Figure 6a—cyan and pink).

The dependence of the mode frequencies on the nanofilm thickness in hybrid Sn trilayers is summarized in Figure 6b. The longitudinal E1g vibrations in both the Ge and Sn layers remain largely insensitive to the increase in the layer thickness (Figure 6b) (black squares and green circles). Meanwhile, the transverse A1g vibrations show opposing trends below the critical film thickness *d* < *d*_crit_, where the Ge modes shift to lower frequencies and the Sn modes shift to higher frequencies as the layer thickness increases; (Figure 6b—red diamonds and pink stars). The insensitivity of the Ge and Sn longitudinal vibrations to film thickness suggests that all of the layers are crystalline. This is in sharp contrast to the behavior observed in Ge–Sn alloys, where both the longitudinal and the transverse modes drastically shift to lower frequencies as the Sn content is increased.^53,54,58,59^ The behavior of the surface phonon mode (SM) (shown as blue inverted triangles and lines in Figure 6a,b) is also unconventional. The SM mode remains at the same energy above the Sn film thickness *d* > *d*_crit_ and moves to higher energies when *d* < *d*_crit_. This behavior cannot be explained by strain and/or amorphous effects that tend to generally decrease vibrational energies.

An in-depth comparison of the Raman peak intensity ratios between the SM mode and the other Sn modes further confirms the existence of a critical Sn nanofilm thickness, *d*_crit_ = 1 nm. The intensity ratio between surface mode *I*(SM) and Sn vibrations *I*(Sn-E1g) and *I*(Sn-A1g) is shown in Figure 6c. Notably, the intensity ratio *I*(SM)/*I*(Sn-E1g) shows a clear regime separation around the critical Sn film thickness *d*_crit_ (Figure 6c) (top graph: green/white circles). In films with thicknesses well below the critical *d* ≪ *d*_crit_, the *I*(SM)/*I*(Sn-E1g) rapidly doubles. In films with thicknesses approaching the critical value *d*_crit_, the intensity ratio shows a local maximum, centered exactly at *d* = *d*_crit_. The intensity ratio *I*(SM)/*I*(Sn-A1g) demonstrates a similar trend (Figure 6c) (bottom graph: pink/white stars). The Raman observations indicate that the hybrid Sn trilayers are unique systems that behave differently from Ge–Sn alloys and glasses, demonstrating important surface phonon mode effects.

### SFSs in Metallic Nanofilms Probed by Impedance Spectroscopy

Complex impedance studies are widely used to determine the capacitance effects of grains and grain boundaries in polycrystalline materials.^60−62^ Generally, it is a technique that can separate the behavior of the material bulk from the grain boundary. More recently, such methods have become more widespread across the 2D community where they have been applied to probe for interfacial and surface effects in ultrathin films.^63^ Thus, complex impedance spectroscopy was chosen as a viable probe to characterize and quantify the effects of SfSs in metallic nanofilms.

**Figure 7:**
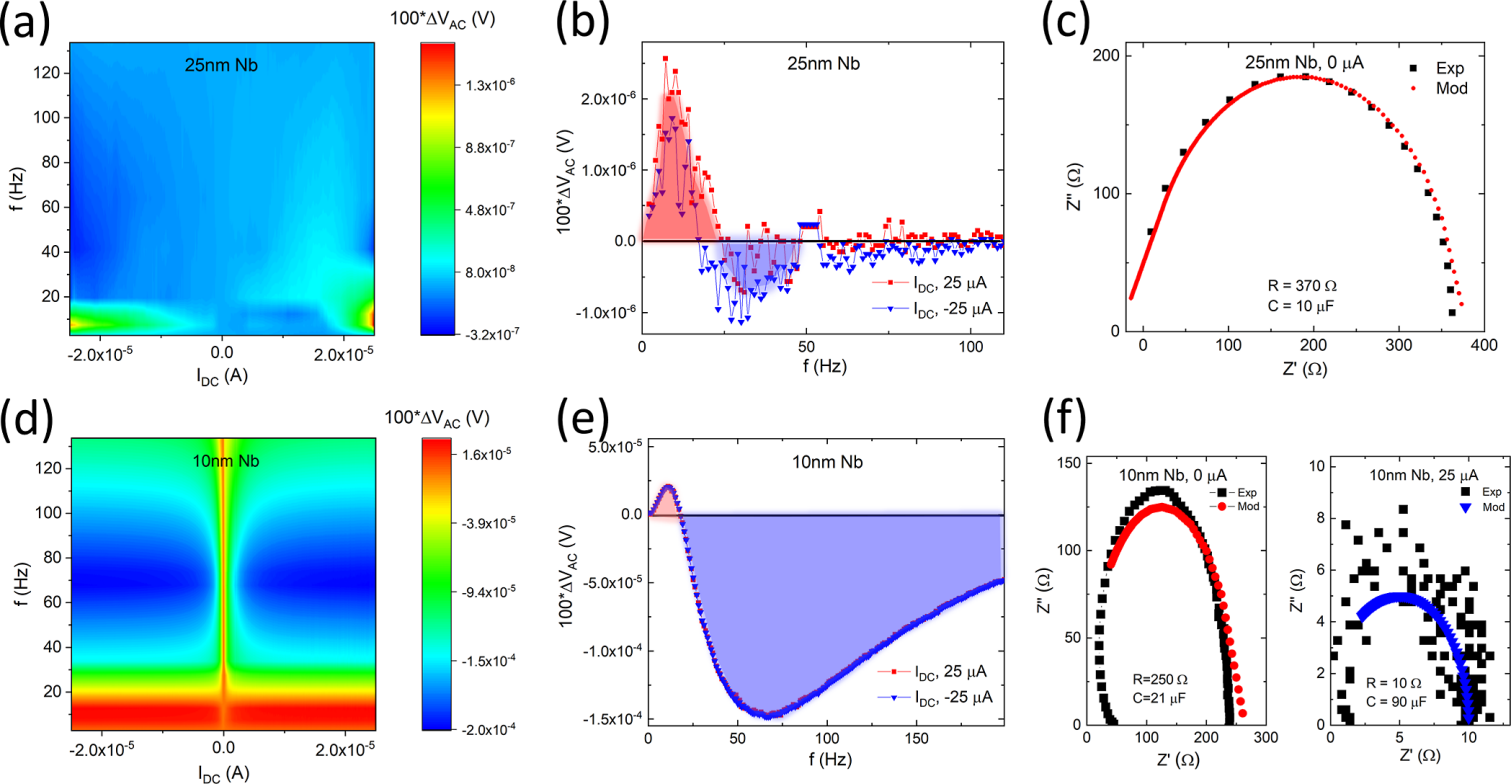
Differential voltage Δ*V*_AC_ and impedance spectroscopy *Z*′, *Z*″ results for Nb films grown on 540 nm thick SiO_2_ oxide: (a,d) Δ*V*_AC_ colormap in the phase space of frequency *f* and DC current *I*_DC_ for 25 and 10 nm thick Nb films, respectively. (b,e) Δ*V*_AC_ frequency linescan at DC current values *I*_DC_ = ± 25 μA for 25 and 10 nm thick Nb films, respectively. (c,f) Impedance spectroscopy Nyquist plot of *Z*′ vs *Z*″ for 25 and 10 nm thick Nb films, respectively. The data is fitted with a single RC model with parameters. All data were collected with AC current *I*_AC_ = 8 nA and frequencies 0 < *f* < 150 Hz. The DC current varied between *I*_DC_ = ± 25 μA.

Impedance spectroscopy was conducted on trilayer samples consisting of Nb films grown on a 540 nm thick SiO_2_ oxide layer deposited onto the n-doped Si substrate. Based on the reflectivity results, the thicknesses of the Nb films were chosen to be 25 and 10 nm, where the latter is the critical thickness *d*_1_ demonstrating zero-reflectivity (see Figure 4b). The differential voltage Δ*V*_AC_ and impedance results were obtained as described in the Methods and Materials section and are summarized in Figure 7. For emphasis, the values of Δ*V*_AC_ were scaled by a factor of 100. The behavior of the differential voltage Δ*V*_AC_ as a function of frequency and DC bias current *I*_DC_ is shown in Figure 7a,d for the 25 and 10 nm thick Nb films, respectively. Markedly, the dependence of the Δ*V*_AC_ on the *I*_DC_ for the 10 nm Nb film is completely symmetrical and invariant with the DC bias direction across all studied frequencies. Moreover, the frequency behavior across the phase space illustrates a monotonic and partially re-entrant transition from positive to negative Δ*V*_AC_ values. This is in stark contrast to the electrical response observed for the 25 nm thick Nb film, where the Δ*V*_AC_ is several orders of magnitude lower and largely negligible at frequencies exceeding *f* > 50 Hz. The effect is most obviously seen by taking frequency linescans of Δ*V*_AC_ at the extreme DC bias currents of *I*_DC_ ± 25 μA for the two studied samples, as depicted in Figure 7b,e. Specifically, the frequency linescans indicate that the frequency dependence for the 10 nm thick Nb trilayer sample is several orders of magnitude greater than in 25 nm thick Nb trilayers and extends to much higher frequencies.

The real and imaginary parts of the impedance for the 25 and 10 nm thick Nb trilayer samples are represented conveniently as a Nyquist plot (see Figure 7c,f). The Nyquist plot has a characteristic semicircular shape as reported in published literature and, accordingly, can be fitted by a parallel RC circuit model.^61,62^ The impedance for the 25 nm thick Nb trilayer can be fitted well with parameters *R* = 370 ± 10 Ω and *C* = 10 ± 5 μF (see Figure 7c). On the other hand, the impedance for the 10 nm thick Nb trilayer requires parameters *R* = 250 ± 10 Ω and *C* = 21 ± 5 μF (see Figure 7f, left panel). The decrease in parameter *R* may be consistent with the presence of the SfS that becomes increasingly dominant as the thickness of the metallic nanofilm is decreased. However, the impedance of the Nb trilayer samples demonstrates most surprising characteristics when combined with a DC bias current *I*_DC_. For the 25 nm thick Nb trilayer, the application of a DC bias current has virtually no effect on the impedance properties and the Nyquist plot remains as shown in Figure 7c and can be fitted with identical RC parameters. However, for the 10 nm thick Nb trilayer, the application of a DC bias current of *I*_DC_ ± 25 μA dramatically shrinks the Nyquist plot, which is mirrored in the new set of RC model parameters (*R* = 10 ± 5 Ω and *C* = 90 ± 5 μF) required to fit the experimental data. The results suggest the presence of a highly mobile, highly polarizable phase in 10 nm thick Nb trilayers which is absent in thicker Nb samples. It is possible that this phase is related to 2DEG contained within the SfSs.

**Figure 8:**
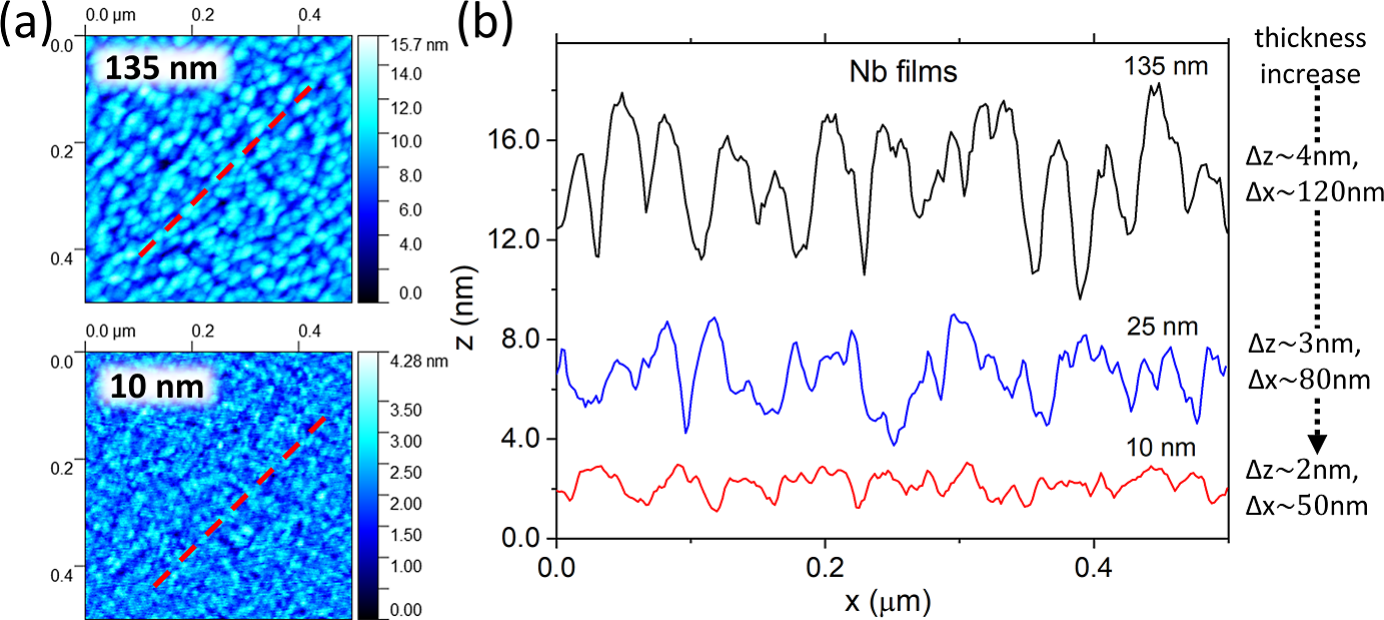
AFM topography of Nb films grown on 540 nm thick SiO_2_ oxide: (a) *z*-directional colormap of 0.5 × 0.5 μm^2^ area for 135 nm (top) and 10 nm (bottom) thick Nb film, respectively (red dashed line indicates the position of *xy*-linescans); (b) *xy*-linescan through a 0.5 μm distance for 135, 25, and 10 nm thick Nb films. Δz variations of the order of ∼2–4 nm are shown.

To conclude the characterization, atomic force microscopy (AFM) experiments were performed on 135, 25, and 10 nm thick Nb trilayers to probe the topography of the thin films, as summarized in Figure 8. The 135 nm thick Nb trilayer was chosen as the bulk control. Figure 8a shows the surface maps for the 135 and 10 nm thick studied Nb samples. As expected, the roughness increases with the thickness of the Nb films. This is most clearly illustrated in Figure 8b, depicting linescan profiles for the studied Nb trilayers. The linescans for the thinner Nb films are shifted vertically for clarity. Here, it is evident that the surface roughness, expressed as parameters δ*z* and δ*x*, signifying out-of-plane and in-plane spatial variations, respectively, increases with surface thickness. For the Nb trilayers with the critical thickness *d*_1_ = 10 nm, the surface roughness is minimal, δ*z* ∼ 2 nm and δ*x* ∼ 50 nm, suggesting a topography composed of semispherical droplet-islands. For comparison, SEM investigation of the topography of all metallic trilayers at the critical metal thickness *d*_1_ is presented in Supporting Information Figure SI2. Notably, no clear relation between surface topography, as probed by AFM and SEM methods, has been established.

## Discussion

The presented experimental reflectivity results for metal nanofilm trilayers combined with extensive modeling have shown that zero reflection occurs under conditions of strongly negative values for ϵ_1_(*d*_1_, λ) and the dominant contribution of the extinction coefficient over the refractive index in *N*_1_(*d*_1_, λ) (see Table 1). These are characteristics of the metallic behavior of the system consisting of the ultrathin metal film and insulating layer which is a Rashba metamaterial or metasurface. In metal nanofilms, due to size quantization, the metallicity is confined to SfSs sandwiching the insulating bulk.^13^ In semiconductors, such as oxides, there are Tamm SfSs, where the electrons are trapped near the surface, and the surface has the same insulating character as the bulk. In nanometer-thick metals, there are Shockley states that are conducting while the bulk states remain insulating. By combining an oxide with a thin metallic film, it may become possible to change the character of SfSs from Tamm type to Shockley type, making the surface electrons mobile and the surface conducting. On the example of trilayers discussed in this paper, the transition from Tamm to Shockley in the SfSs or the MIT transformation may be tuned by varying the thickness of the metal nanofilm, resulting in striations of the cross-sectional electronic density. That density variation may result in the creation of a Rashba metamaterial or metasurface.

In the published literature, stratified media have shown promising antireflective properties. Specifically, it was demonstrated that the interface of two dielectrics can be made highly transparent (transmittance ∼70%) by the addition of a metal overcoat containing density discontinuities with thicknesses ∼60–300 nm.^64^ Similarly, one-dimensional plasmonic metafilms based on periodic arrays of metal and dielectric regions have been explored theoretically and confirm low reflection across specific frequency ranges.^65^ Notably, metal-based nanostructures have shown tunable reflective camouflage properties.^66^ Our findings that trilayers can be tuned toward Rashba metamaterials or metasurfaces sustaining zero reflectivity and perfect darkness is a progressive leap in this direction.

The discovered phenomenon of zero reflectivity occurs only at specific critical thicknesses *d*_1_ for the metal nanofilms, ranging from *d*_1_ = 1.0 nm to *d*_1_ = 10.0 nm in the Ge/Sn and Nb trilayers, respectively. The critical thickness *d*_1_ is evident in other probes, such as AFM or Raman and impedance spectroscopy. For example, the Raman studies on the Ge/Sn trilayers reveal a regime change as the critical thickness is approached (*d* → *d*_1_ = 1.0 nm) and local relative maxima in the surface mode (SM) intensity ratio at *d* = *d*_1_ (see Figure 6). The results imply that the surface mode is slightly enhanced at the critical thickness *d* = *d*_1_ in these structures, which may signify a strengthening in coherence of the SfSs—tuned by varying the thickness of the metal nanofilm. In films with thickness well below the critical *d* < *d*_1_ the SfSs may be too disparate to be cohesive, whereas for *d* > *d*_1_, the SfSs become overwhelmed by bulk modes. The fact that the reflectivity increases as the film thickness deviates from *d*_1_ in either direction (see, Figures 2 and SI1) further confirms that the SfSs play a key role in the observed optical phenomenon.

To evaluate the surface topography, the AFM investigation of the Nb trilayers has shown that the average surface roughness decreases as the film thickness decreases (see Figure 8). The surface topography for Nb trilayers at the critical thickness *d*_1_ = 10.0 nm consists of semiregular island-like droplets of average height ∼2 nm and average diameter ∼50 nm, see Figure 8b. Although it is difficult to ascertain the importance of surface topography in optical reflectivity, its role may be significant for the cohesiveness of the SfSs and their percolation through the polycrystalline sample as well as the Rashba field effects. A comparative SEM study of the metallic trilayers at the critical thickness d_1_ has not revealed any significant similarities in the topography of the materials (see Figure SI2 in the Supporting Information).

Impedance studies on the Nb trilayers have revealed a remarkable transformation in the electronic character at the critical thickness *d*_1_ = 10.0 nm see, Figure 7. At *d* = *d*_1_, the electrical conductivity of the Nb nanofilms increases by several orders of magnitude in a DC current bias and depends very strongly on the excitation frequency (see Figure 7e,f). This has not been observed in thicker Nb films (*d* = 25 nm). The behavior suggests that at the critical thickness *d*_1_, the SfSs become coherent enough to sustain a form of 2DEG which is highly mobile and is easily polarizable by any applied DC bias, while the bulk of the Nb nanofilm remains insulating (see Figure 7f). Speculatively, at the critical thickness, the 2DEG contained within the SfSs may be tuned toward MIT by the surface Rashba field and harbor novel phases, such as charge-cluster states.^14,21^ The substantial increase in the capacitance of the Nb nanofilm (*C* = 90 μF) at the critical thickness d_1_ under an I_DC_ bias may evidence the formation of charge-cluster phases to corroborate the premise. In any case, our results illustrate that the presence of the 2DEG in the SfSs of the Nb nanofilms is very closely linked to the observed phenomenon of zero reflectivity and suggests that similar effects are expected to occur in all metallic nanofilms, underlying the universality of the approach. In the case of Bi and Ge/Sn trilayers, the 2DEG may acquire additional topological character.

The inclusion of graphene has frequently lead to an increase in the antireflective and transmissive properties of a material, as was demonstrated by combining monolayer graphene with a photonic crystal resonator made up of multilayer dielectric (or Bragg mirrors) to theoretically achieve an absorption of 100% in the near-infrared and visible light spectrum through critical coupling.^67^ Similarly, the integration of graphene into a waveguide-based optical modulator has been shown to substantially increase its transmittance.^9,10^ Furthermore, the SfSs may play a key role in semiconducting nanofilms as well, manifesting as Shockley and Tamm states. Recently, ZnO films with thicknesses ∼100–120 nm have shown antireflective properties in the THz regime.^68^ Thus, the effect of 2D materials and systems where SfSs dominate the optical responses in complex multilayer structures may be extremely broad, providing a convenient mechanism for properties’ control and design.

## Conclusions

To conclude, we have developed a novel approach to create RMMs with zero light reflectivity by tuning the thickness of the metal nanofilm in trilayers, composed of a combination of oxides and a nanometer-size metal film. We demonstrate that the RMMs’ transformation in the studied trilayer structures likely arises due to a potential MIT in the SfS, similar to those found in the 2DEG. The novel RMMs are characterized by negative permittivity components in the dielectric tensor and Veselago lens properties, which leads to an unprecedented zero reflectivity phenomenon across a broad range of frequencies and angles of the incident light. The RMMs comprise a novel class of materials with size effects in any dimension, where the optoelectronic properties are dictated by the SfS. Our phenomenological approach to create RMMs opens up avenues to achieve surfaces with zero reflectivity practically for any desired frequency range and any angle. The present work and its findings should facilitate new developments and advances in multiple sectors and industries working to minimize light reflection.

## Experimental Section

### Materials

The metallic nanofilms were grown on top of an oxide layer using the electron beam deposition technique. Metals such as Sn, Ag, Au, Pt, Bi, and Nb were used. For Sn metallic films, the inclusion of an additional Ge wetting buffer layer, 1 nm thick, improved the quality of the nanofilms. The oxide layers SiO_2_ and Al_2_O_3_, with varying thicknesses of 540–60 nm, were used. The oxide layers were grown using the electron beam deposition technique on an n-doped Si substrate.

### Reflectivity Studies

The reflectivity was measured using a commercial spectroscopic ellipsometer with a quartz tungsten halogen light source covering the frequency range of 400–1000 nm at room temperature. The angle of incidence for ellipsometry experiments was varied between 0 and 90 deg.

The refractive index and the optical constants of the nanofilms were obtained via the transfer matrix methods (as described in detail in the Supporting Information). The dependence of the metal nanofilm thickness *d*_1_ on the effective refractive index *N*_eff_ takes the form
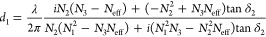
2where *N*_1_, *N*_2_, and *N*_3_ are the optical constants of the metal nanofilm, the oxide layer, and the Si substrate, respectively. δ_1_ = 2π*N*_1_*d*_1_/λ and δ_2_ = 2π*N*_2_*d*_2_/λ are the phase parameters associated with the metal nanofilm and the oxide and *d*_1_ and *d*_2_ are their respective thicknesses.

The behavior of the dielectric function is given by ϵ_1_(*d*,λ) = *N*_1_^2^. At the interference minimum, where zero reflectivity is observed, *N*_eff_ = 1 + 0*i*, the dielectric function simplifies to
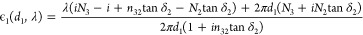
3where *n*_32_ = *N*_3_/*N*_2_. Notably, the only nonconstant variables here are the light wavelength λ and the thickness of the metal film *d*_1_. Taking the square root of the dielectric constant ϵ_1_(*d*_1_, λ) gives the refractive and the extinction coefficients of the metal nanofilm, *N*_1_ = *n*_1_ + *ik*_1_, at locations where zero reflectivity is achieved.

### Raman Spectroscopy

Raman spectroscopy was performed using a Horiba Raman spectrometer with a red He–Ne laser of wavelength λ = 632.8 nm. Diffraction grating of 600/mm was sufficient to produce high resolution in the experiments. The laser power and acquisition time varied between 0 and 10% of the nominal power 3.5 mW and 1–10 s, respectively, to prevent sample overheating effects and peak and background broadening. The laser beam was focused to a spot size of 1 μm on the surface of the samples. The Raman peaks were fitted using the Lorentzian form to determine peak position and full width half-maximum (fwhm).

### Atomic Force Microscopy

The AFM surface topography results were obtained using a Park XE7 Park Atomic Force Microscope Park XE7 system. The data was obtained with magnetic PPP-MFMR Nanosensors AFM tips with torque constant *T* = 2.8 N/m and at frequencies *f* = 75 kHz. The tips have lateral resolution considerably higher than 50 nm. Area scans of 0.5 × 0.5 μm^2^ and linescans of 0.5 μm produced the highest-resolution images.

### Surface Impedance Spectroscopy

Differential AC conductance and complex surface impedance studies in Nb nanofilms were performed using a setup consisting of a Standford Research SR830 lock-in amplifier, a Keithley 6221 current source, and a room temperature transformer. The lock-in amplifier was used to generate a sinusoidal output voltage *V*_sin_(*f*) ∼ 0.008 V. The sine wave reference signal was then passed across a fixed resistor *R* ∼ 1 MΩ, converting it to an AC current of magnitude *I*_AC_(*f*) = 8 nA with a particular frequency *f*. The Nb nanofilm was contacted with gold (Au) wire of 25 μm diameter in a standard linear 4-probe configuration. Silver paint was used to provide good electrical contact between the Au wire and the bilayer surface. The Au wires were reinforced externally with an Araldaite rapid epoxy for mechanical stability. The AC current *I*_AC_ was passed across the outer two electrodes of the Nb nanofilm, and an AC voltage *V*_AC_ was picked up across the inner two electrodes. A DC bias current *I*_DC_ was sent through the sample in series with the AC current. The bias DC current ranged around *I*_DC_ ± 25 μA. To normalize all signals, the voltage value obtained at zero bias DC current *I*_DC_ = 0A was subtracted to obtain the differential AC voltage Δ*V*_AC_. The differential AC voltage signal Δ*V*_AC_ was passed through a room temperature transformer with a gain *G* ∼ 100 to enhance the signal-to-noise ratio and reduce noise. The differential AC voltage Δ*V*_AC_ is inversely proportional to the differential conductance ∼d*I*/d*V*. The differential AC voltage was measured at a fixed AC frequency *f*, where the DC bias current *I*_DC_ was varied in small increments. Several frequencies in the range 0 < *f* < 150 Hz were investigated.

Similarly, differential AC voltage sweeps as a function of frequency *f* at a fixed DC bias current were conducted. In a frequency sweep, the *V*_AC_ values at nonzero bias *I*_DC_ were normalized by subtracting the values at *I*_DC_ = 0A for each frequency. The impedance spectroscopy studies were conducted with zero bias DC current *I*_DC_ = 0A and a fixed value of AC current *I*_AC_(*f*) = 8 nA while varying the frequencies in small increments between 0 < *f* < 150 Hz. The lock-in amplifier collected both the in-phase and out-of phase contributions of the *V*_AC_ signal – *V*_AC_(in) and *V*_AC_(out). Real and imaginary parts of the complex impedances were then calculated as *Z*′ = *V*_AC_(in)/(100 × *I*_AC_) and *Z*″ = *V*_AC_(out)/(100 × *I*_AC_), respectively. This method for studying complex impedances was tested previously in the literature and has been seen to agree with results from conventional impedance analyzers in the low frequency range up to ∼300 Hz^69^.^70^ A particular advantage of the lock-in amplifier setup is that it is considered to be a less invasive technique, even suitable for biological specimens.^69^ Using this preferred method for determining the complex impedance in the system ensured that the Nb nanofilm was not irreversibly or destructively affected in the course of these studies. The complex impedance results were simulated by a conventional RC-model, where a resistor *R* and a capacitor *C* are connected in parallel. In this manner, the real and imaginary parts of the complex impedance are given by
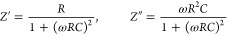
4where *R* and *C* are the resistor and capacitor, respectively, and ω is the excitation frequency ω = 2π*f*, as detailed in the literature.^60^

## Supporting Information Available

Reflectivity results of the Ge/Sn trilayers above the critical thin film thickness; SEM images of the trilayers at critical metal nanofilm thickness; and theoretical derivation of the targeted optical constant and the thickness of the coating layer (PDF)
