# Supplementary material for: Rashba Metamaterials and Metasurfaces with Zero Reflectivity and Effect of Surface States in Ultrathin Metal Films
Source: ACS Appl Mater Interfaces. 2024 Jan 17;16(4):4904–17. doi: 10.1021/acsami.3c15285 (PMC10835661; doi:10.1021/acsami.3c15285)
Supplement: Supplementary file 1 — am3c15285_si_001.pdf [file am3c15285_si_001.pdf]

Supporting information: Rashba metamaterials and metasurfaces with zero reflectivity, effect of Surface States in ultra-thin metal films

Fedor Kusmartsev*
College of Art and Science, Khalifa University, PO Box 127788, Abu Dhabi, UAE
Microsystem and Terahertz Research Center, Chengdu 610200,P.R. China and
Department of Physics, Loughborough University, Loughborough LE11 3TU, United Kingdom

Binglei Zhang, Yang Liu, and Yi Luo
Microsystem and Terahertz Research Center, Chengdu 610200,P.R. China

James Vincent-Ward and Anna Kusmartseva†
Department of Physics, Loughborough University, Loughborough LE11 3TU, United Kingdom

Fatemah Alkallas and Amira Ben Gouider Trabelsi
Department of Physics, College of Science, Princess Nourah bint
Abdulrahman University, P.O. Box 84428, Riyadh 11671, Saudi Arabia
(Dated: January 1, 2024)

The Supporting Information contains: Reflectivity results of the Ge/Sn tri-layers above the critical thinfilm thickness; SEM images of the metallic tri-layers at critical thinfilm thickness; theoretical derivation of the targeted otpical constant and the thickness of the coating layer (PDF).

REFLECTIVITY OF SURFACES COATED BY DIFFERENT NANOMETER THICK METAL FILMS

A general method has been discovered that allows to reduce the reflectivity of a trilayer system, consisting of a substrate, an oxide and a thin metallic layer to nearly or completely zero R=0. The approach utilises the high conductivity and topological properties of the surface states in ultra-thin metal films to improve the quality factor of the Fabry Perot interferometer effects of the oxide interlayer deposited on the substrate. Zero reflectivity or near zero reflectivity has been demonstrated for many different metals - in particular Bi, Nb, Ag, Pt, Ge/Sn. The Bi, Nb and Ag films have been grown on a 540 nm thick SiO_2 oxide layer. The Pt films have been grown on a thinner 430 nm thick SiO_2 layer. Ge/Sn films have been grown on 60 nm thick Al_2O_3. The oxide plays the role of a Fabry Perot (FP) resonator. The reflectivity results for Sn, Bi, Nb, Ag and Pt are summarised in the main paper.

Tri-layer samples containing Sn nano-films were deposited onto a Ge layer grown on top of 60 nm Al_2O_3 oxide (or sapphire) on an n-doped Si substrate. Here, the Ge layer was acting as a wetting buffer layer ensuring that the Sn spread more homogeneously around the Al_2O_3 surface. To produce highest quality films equal thicknesses of Ge and Sn were used. Samples with various Ge/Sn thicknesses 0.3/0.3, 0.5/0.5, 0.8/0.8, 1.0/1.0, 1.2/1.2 nm and 0.8/1.2 nm have been fabricated. The main paper shows the reflectivity results for Ge/Sn tri-layer samples with thicknesses 0.3 - 1.0 nm. The complete reflectivity study is shown in Fig. S1. Reflectivity is seen to increase as the thickness of the Ge/Sn layers exceeds the critical value d_{crit}=1 nm.

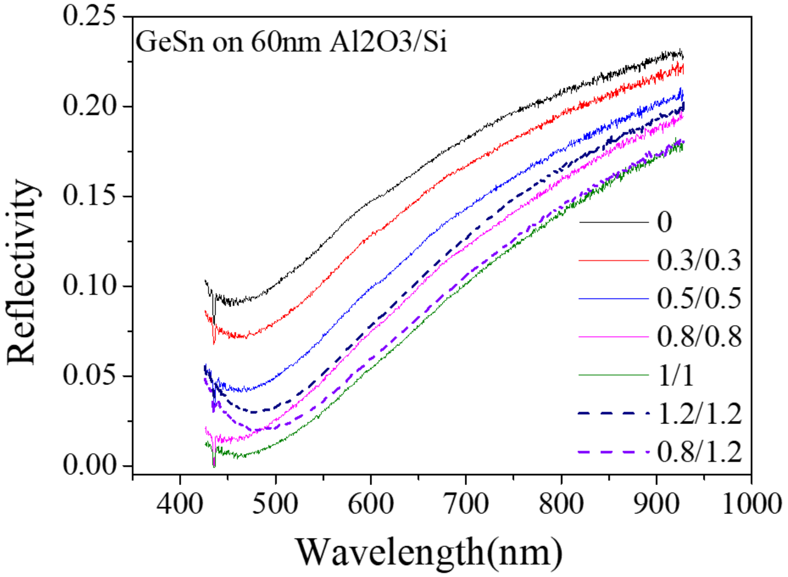

FIG. S1: Left panel: The reflectivity results for Ge/Sn devices over the visible light frequency range. The wavelengths are given in nm. The studied Ge/Sn film thicknesses were equal to 0/0 nm (black), 0.3/0.3 nm (red), 0.5/0.5 nm (blue), 0.8/0.8 nm (purple), 1.0/1.0 nm (green), 1.2/1.2 nm (navy dash), 0.8/1.2 nm (purple dash), respectively. The broad minimum (in all curves) around 450 - 460 nm corresponds to the quarter wavelength destructive interference in the Fabry Perot resonator(FPR) made from 60nm Al_2O_3 oxide layer deposited onto a n-doped Si substrate. Note, that its $\lambda/4$-wavelength remains the same or decreases with the thickness (see, the pink curve). At 430 - 440 nm, there is a further significant decrease in reflectivity.

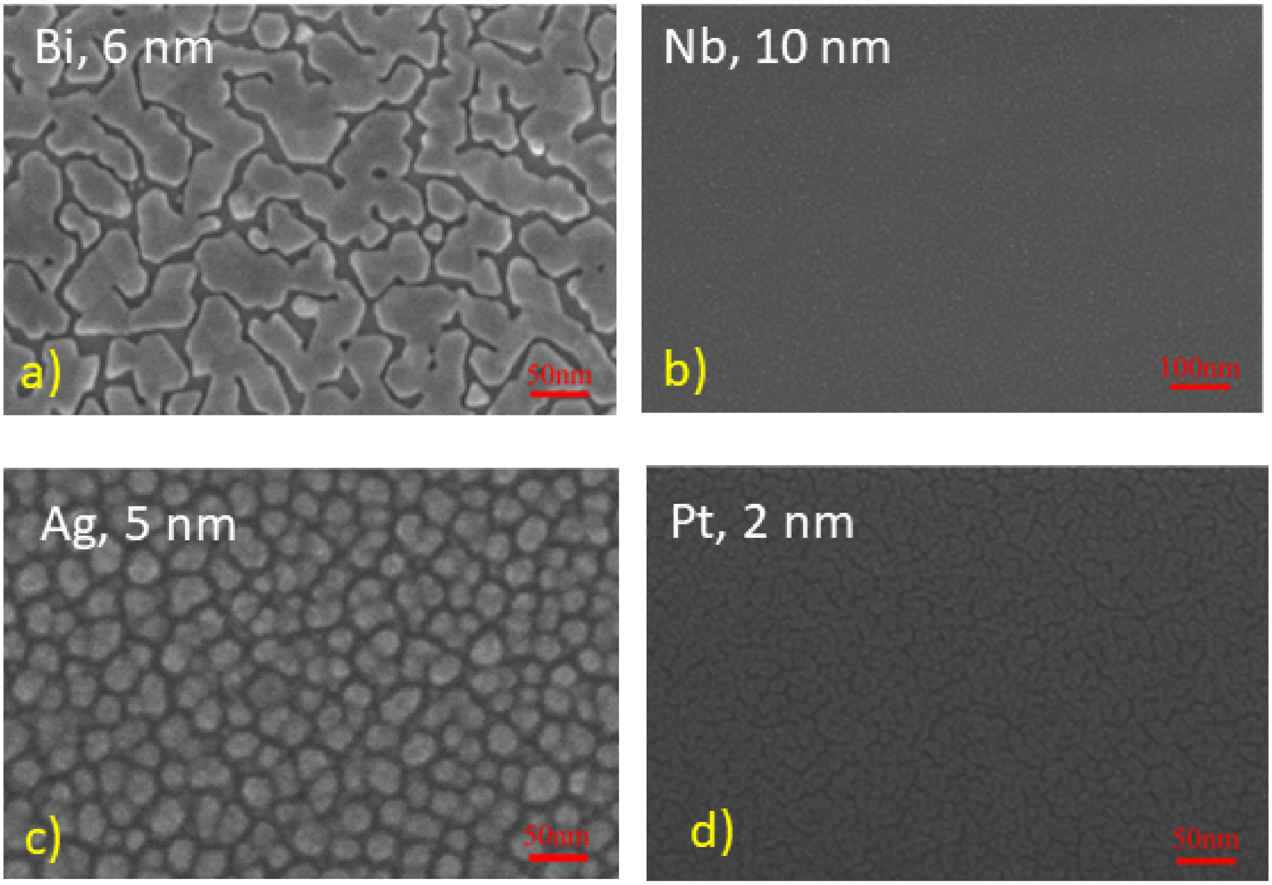

FIG. S2: SEM images for the metallic trilayer at the critical thickness d_{crit}. a) Bi trilayers with thickness d_{crit}=6 nm; b) Nb trilayers with thickness d_{crit}=10 nm; c) Ag trilayers with thickness d_{crit}=5 nm; d) Pt trilayers with thickness d_{crit}=2 nm;

SEM images of the metallic nanofilms

We have conducted SEM experiments to study the topography of the metallic trilayers at the critical thickness d_{crit}, see Fig. S2. Notably, the SEM images illustrate that the topography is vastly different for the investigated metallic trilayers. The Bi trilayers at d_{crit} show large percolated pathways on the surface, see Fig. S2a). The Nb trilayers at d_{crit} show a tiny granular surface, see Fig. S2b). The Ag trilayers surface at d_{crit} is made up of large granules interconnected by small granules, see Fig. S2c). Finaly, the Pb trilayers at d_{crit}, show a surface with very shallow percolated paths, see Fig. S2d). Therefore, the relation between structural topography and the zero reflectivity phenomena observed in these systems is not trivial or straightforward. Although, given that the critical thicknesses d_{crit} are significantly different for the various metallic trilayer, ranging from 2 nm (Pt) to 10 nm (Ng) it is somewhat unsurprising that their topographies are so distinct.

DERIVATIVE OF THE TARGETED OPTICAL CONSTANTS AND THICKNESS OF COATING LAYER

The experimental results in the previous section have shown that the optical reflectivity is determined by the effective refractive index on the surface. The effective refractive index can be tuned by combining different materials and by varying the thickness of the metal in the trilayer systems. The next section demonstrates the relationship between the effective refractive index of the trilayer N_{eff} and the thickness of the metal layer d_1 .

The dependence of the nano-film thickness d_1 on the effective refractive index N_{eff}

The trilayer systems may be considered as a Fabry Perot interferometer that has been coated by a nano-meter thick metal layer as shown in Fig. 1 in main text. The reflectance of the trilayer surface is calculated by the transfer matrix method. The following equation determines the relationship between the electric field and the magnetic field on the surface:

$$\begin{pmatrix} E_0 \\ H_0 \end{pmatrix} = \begin{bmatrix} cos\delta_1 & isin\delta_1/\eta_1 \\ i\eta_1 sin\delta_1 & cos\delta_1 \end{bmatrix} \begin{bmatrix} cos\delta_2 & isin\delta_2/\eta_2 \\ i\eta_2 sin\delta_2 & cos\delta_2 \end{bmatrix} \begin{pmatrix} E_s \\ H_s \end{pmatrix} , \tag{S1}$$

where $\delta = 2\pi N d cos\theta_r/\lambda$, $\eta = \gamma N cos\theta_r$ for TE wave, $\eta = \gamma N/cos\theta_r$ for TM wave and $\gamma = \sqrt{(\varepsilon_0/\mu_0)}$. Here $E_0(E_s)$ and $H_0(H_s)$ are electric and magnetic fields on the surface(substrate) of the tri-layer, respectively. N is the complex index of refraction ($N = n + ik$) and d is the thickness. θ_r is the refractive angle, which is determined by Snell's law The subscripts $1, 2$ represent the metallic and the oxide layer respectively, the subscripts 0 and s represent the surface and the substrate. The reflectance is calculated by $R = \rho\rho^*$ and ρ is the reflection coefficient:

$$\rho = \frac{N_0 - N_{eff}}{N_0 + N_{eff}}, \tag{S2}$$

where $N_{eff} = H_0/(\gamma E_0)$. For normal incidence of light the parameter θ can be neglected. For all considered tr-ilayer systems the wavelength at which reflectivity minima occur is greater than 400nm. The parameters associated with the zero reflectivity conditions in different trilayers are summarised in Tab. 1 of the main paper.

For a specific value of N_{eff}, the thickness (d_1) of the metal layer shall be determined. Considering that the thickness of the metal layer ($\sim 1nm$) is much smaller than the interested wavelength ($> 400nm$) and the optical constant N is also a small number, the following approximations may be used $\delta_1 \ll 1$, $sin\delta_1 \approx \delta_1$ and $cos\delta_1 \approx 0$ to simplify the calculations. Thus, the transfer matrix M can be rewritten in the following form

$$\begin{aligned} M &= \begin{bmatrix} cos\delta_1 cos\delta_2 - \frac{\eta_2}{\eta_1} sin\delta_1 sin\delta_2 & icos\delta_2 sin\delta_1/\eta_1 + icos\delta_1 sin\delta_2/\eta_2 \\ i\eta_1 cos\delta_2 sin\delta 1 + i\eta_2 cos\delta_1 sin\delta 2 & cos\delta_1 cos\delta_2 - \frac{\eta 1}{\eta 2} sin\delta_1 sin\delta_2 \end{bmatrix} \\ &\approx \begin{bmatrix} cos\delta_2 - \frac{\eta_2}{\eta_1}\delta_1 sin\delta_2 & icos\delta_2\delta_1/\eta_1 + isin\delta_2/\eta_2 \\ i\eta_1 cos\delta_2 \delta 1 + i\eta_2 sin\delta 2 & cos\delta_2 - \frac{\eta 1}{\eta 2}\delta_1 sin\delta_2 \end{bmatrix} \\ &= \begin{bmatrix} cos\delta_2 & isin\delta_2/\eta_2 \\ i\eta_2 sin\delta 2 & cos\delta_2 \end{bmatrix} + \begin{bmatrix} -\frac{\eta_2}{\eta_1} sin\delta_2 & icos\delta_2/\eta_1 \\ i\eta_1 cos\delta_2 & -\frac{\eta 1}{\eta 2} sin\delta_2 \end{bmatrix} \delta_1 = M_2 + P\delta_1 \end{aligned} \tag{S3}$$

Recalling Eq.S1, N_{eff} is further simplified and becomes:

$$N_{eff} = \frac{n_1(n_2 n_3 cos\delta_2 + in_2^2 sin\delta_2) + n_1^2(in_2 cos\delta_2 - n_3 sin\delta_2)\delta_1}{n_1(n_2 cos\delta_2 + in_3 sin\delta_2) + n_2(in_3 cos\delta_2 - n_2 sin\delta_2)\delta_1}\gamma , \tag{S4}$$

$$N_{eff} = \frac{iN_1 \cos\delta_2 \sin\delta_1 + iN_2 cos\delta_1 \sin\delta_2 + N_S\left(\cos\delta_1\cos\delta_2 - N_1/N_2 \sin\delta_1 sin\delta_2\right)}{\cos\delta_1\cos\delta_2 - N_2/N_1 \sin\delta_1\sin\delta_2 + iN_S\left(\cos\delta_2\sin\delta_1/N_1 + \cos\delta_1\sin\delta_2/N_2\right)} . \tag{S5}$$

By solving the equation one can obtain the dependence of the thickness d_1 of the metal layer on the effective optical constant n_{eff}:

$$d_1 = \frac{\lambda}{2\pi N_1}\delta_1 = \frac{in_1 n_2(n_3 - n_{eff})cos\delta_2 + n_1(-n_2^2 + n_3 n_{eff})sin\delta_2}{n_2(n_1^2 - n_3 n_{eff})cos\delta_2 + i(n_1^2 n_3 - n_2^2 n_{eff})sin\delta_2}\frac{\lambda}{2\pi N_1} . \tag{S6}$$

Derivation of the typical form of N_{eff}

N_{eff} can be obtained by substituting $\delta_1 = \frac{2\pi N_1 d_1}{\lambda}$ into Eq.S4 and dividing the numerator and denominator by $n_2(in_3 cos\delta_2 - n_2\sin\delta_2)\frac{2\pi N_1}{\lambda}$ simultaneously, giving the expression:

$$\begin{aligned} N_{eff} &= \left(-I\frac{\lambda}{2\pi} + \frac{n_1^2(n_2cos\delta_2 + in_3sin\delta_2)}{n_2(n_3cos\delta_2 + in_2sin\delta_2)}d_1\right) / \left(\frac{(n_2cos\delta_2 + in_3sin\delta_2)\lambda}{i2\pi n_2n_3cos\delta_2 - 2\pi n_2^2sin\delta_2} + d_1\right) \\ &= \frac{C_2 + C_3d_1}{C_1 + d_1} = \frac{c_2 i + c_3e^{i\phi_3}d_1}{c_1e^{i\phi_1} + d_1} \ , \end{aligned} \tag{S7}$$

where $c_2 = -I\frac{\lambda}{2\pi}$. c_1, ϕ_1 and c_3, ϕ_3 are determined by the correspondence of $c_1e^{i\phi_1} = C_1 = \frac{(n_2cos\delta_2+in_3sin\delta_2)\lambda}{i2\pi n_2n_3cos\delta_2-2\pi n_2^2sin\delta_2}$, $c_3e^{i\phi_3} = C_3 = \frac{n_1^2(n_2cos\delta_2+in_3sin\delta_2)}{n_2(n_3cos\delta_2+in_2sin\delta_2)}$. Thus, the quantities become

$$c_1 = |C_1| \ , \tag{S8a}$$

$$\phi_1 = ArcTan\left(Im(C_1)/Re(C_1)\right) \ , \tag{S8b}$$

$$c_3 = |C_3| \ , \tag{S8c}$$

$$\phi_3 = ArcTan\left(Im(C_3)/Re(C_3)\right) \ , \tag{S8d}$$

where $|C|$ represents the norm of the complex number C, Im and Re represent the imaginary and real parts of the content.

REDUCTION OF GSE TO SOME RELEVANT FORMS

One can note that equation (S1) is system of equations. It has a general forms although some components are still missing. Below we will discuss those forms of NLS which found direct applications in different areas physics, which covers not only solitons[1][2] but other phenomena such as self-trapping[3][4],[5] and plasma caviton formation[6][7]. Here we consider two solitons coupled to each other. In one case, we obtain the vector nonlinear Schrödinger equation (VNSE). In the other case, the scalar nonlinear Schrödinger equation (SNSE) is obtained. The difference between VNSE and SNSE is that the former involves coupled solitons and the latter involves decoupled solitons.

* Corresponding author: fedor.kusmartsev@ku.ac.ae
† Corresponding author: A.Kusmartseva@lboro.ac.uk

[1] Kusmartsev F. On Classification of Solitons. Physica Scripta. 1984;29(1):7.
[2] Kusmartsev FV. Application of Catastrophe Theory to Molecules and Solitons. Physics Reports. 1989;183(1):1-35.
[3] Kusmartsev F, Rashba E. Self Trapping from Degenerate Bands (spin S= 1) and Related Phenomena. Harvard Library. 1984.
[4] Kusmartsev F, Rashba E. Jahn-Teller Symmetry Breaking of an Autolocalized Barrier. ZhETF Pisma Redaktsiiu -JETP Lett. 1981;33:164.
[5] Kusmartsev F. Self-trapping in Random Nonhomogeneous Media. Physics Letters A. 1987;121(2):71-6.
[6] Kusmartsev F, Rashba E. Self-Trapping in Crystals and Nonlinear Wave Processes: Self-Trapping Barrier for Plasma Caviton. Zh Eksp Teor Fiz - JETP. 1983;84:2064.
[7] Kusmartsev F, Rashba E. Zh. eksper. teor. Fiz., Pisma 37, 106 (1983); CAS Soviet Phys. J exper theor Phys, Letters-JETP Lett. 1983;37:106.
